# Adrenergic Signaling: A Targetable Checkpoint Limiting Development of the Antitumor Immune Response

**DOI:** 10.3389/fimmu.2018.00164

**Published:** 2018-02-06

**Authors:** Guanxi Qiao, Minhui Chen, Mark J. Bucsek, Elizabeth A. Repasky, Bonnie L. Hylander

**Affiliations:** ^1^Immunology, Roswell Park Comprehensive Cancer Center, Buffalo, NY, United States

**Keywords:** adrenergic, norepinephrine, antitumor immune response, temperature, stress, β-blocker

## Abstract

An immune response must be tightly controlled so that it will be commensurate with the level of response needed to protect the organism without damaging normal tissue. The roles of cytokines and chemokines in orchestrating these processes are well known, but although stress has long been thought to also affect immune responses, the underlying mechanisms were not as well understood. Recently, the role of nerves and, specifically, the sympathetic nervous system, in regulating immune responses is being revealed. Generally, an acute stress response is beneficial but chronic stress is detrimental because it suppresses the activities of effector immune cells while increasing the activities of immunosuppressive cells. In this review, we first discuss the underlying biology of adrenergic signaling in cells of both the innate and adaptive immune system. We then focus on the effects of chronic adrenergic stress in promoting tumor growth, giving examples of effects on tumor cells and immune cells, explaining the methods commonly used to induce stress in preclinical mouse models. We highlight how this relates to our observations that mandated housing conditions impose baseline chronic stress on mouse models, which is sufficient to cause chronic immunosuppression. This problem is not commonly recognized, but it has been shown to impact conclusions of several studies of mouse physiology and mouse models of disease. Moreover, the fact that preclinical mouse models are chronically immunosuppressed has critical ramifications for analysis of any experiments with an immune component. Our group has found that reducing adrenergic stress by housing mice at thermoneutrality or treating mice housed at cooler temperatures with β-blockers reverses immunosuppression and significantly improves responses to checkpoint inhibitor immunotherapy. These observations are clinically relevant because there are numerous retrospective epidemiological studies concluding that cancer patients who were taking β-blockers have better outcomes. Clinical trials testing whether β-blockers can be repurposed to improve the efficacy of traditional and immunotherapies in patients are on the horizon.

## Introduction

Psychosocial and physical stresses have long been believed to negatively affect health and reduce our resistance to immune-mediated diseases (including cancer), but the mechanisms have been poorly understood (1, 2). A comprehensive review of early work on the effects of stress on tumor growth in animal models highlights the dichotomous results between viral tumors which grow faster with stress and non-viral/chemically induced tumors which grow more slowly during the stress period (3). In the last decade or so, however, we are beginning to understand the myriad mechanisms by which chronic adrenergic stress molds the immune response and promotes tumor growth (2–9). In this brief review, we will first give examples of how adrenergic signaling impacts immune cells and then how it affects tumor growth. We then summarize how preclinical mouse models are used to study the effects of different stress types on tumor growth, survival, and metastasis, highlighting the idea that tumors attract their own innervation in a process akin to angiogenesis. We emphasize the potential ramifications of the baseline cold stress which is imposed on mice by sub-thermoneutral housing and how this impacts our understanding of the capabilities of the endogenous immune response. In addition, in light of recent excitement about the potential of new immunotherapies to treat cancer, we discuss how adrenergic suppression of the antitumor immune response is a targetable checkpoint which may be blocked to increase the efficacy of immunotherapy. Finally, we summarize retrospective clinical epidemiological studies that support the idea of “repurposing” β-blockers for use in oncology.

## Neural Regulation of the Immune Response

Stress can impact the immune response through two major neural pathways, the hypothalamic/pituitary/adrenal (HPA) axis and the sympathetic nervous system (SNS); we have recently reviewed these pathways in depth (10). While stimulation of the HPA axis results in the release of glucocorticoids from the adrenal cortex, the SNS innervates the adrenal medulla and stimulates release of the catecholamine neurotransmitters, epinephrine (Epi) and, to a lesser extent, norepinephrine (NE) (11). Perhaps more importantly for the purpose of this review, NE is also released by postganglionic sympathetic neurons which densely innervate both primary and secondary lymphoid organs (12–15) as well as essentially all other organs. Adrenergic receptors (ARs) for NE and Epi are located on the surface of most cells, including immune cells, and thus in addition to the regulatory influences of chemokines and cytokines, NE released from sympathetic nerve endings also plays a critical role in regulating immune cells. In fact, we are now realizing that neural reflexes may reach immune cells in a fraction of the time (milliseconds) that it takes chemokines and cytokines to circulate and have an effect on these cells (16). While activation of the SNS in response to acute stress such as exercise or injury promotes a rapid immune response (17), chronic ongoing stress often is immunosuppressive (18). As discussed below in the Section “Modeling the Tumor-Promoting Effects of Adrenergic Stress/Signaling in Mice”, suppression of the antitumor immune response is one of the consequences of this chronic stress.

Adrenergic receptors are a class of G protein-coupled receptors and are subdivided into two types (α and β) based on their structure, pharmacology, and signaling mechanisms, and these include several subtypes (19). Although there are many studies showing that both α- and β-ARs are expressed by innate immune cells, including neutrophils (20, 21), monocytes (22–24), macrophages (22, 24–26), mature DC (22), NK cells (27–33), and hematopoietic stem cells and progenitors (34), β2-ARs are the most highly expressed subtype on both innate and adaptive immune cells. T and B cells exclusively express β2-AR (35–37); this is also true for hematopoietic stem cells and progenitors (34). Consequently, β_2_-ARs are regarded as the main mediators of the immune effects of catecholamines (8). Activation of these receptors activates adenylate cyclase to increase intracellular cAMP, which in turn activates PKA which ultimately activates downstream transcription factors (2, 35, 36, 38–40). The details of this signaling pathway have recently been reviewed for both immune cells (41) and cancer cells (9).

### Adrenergic Regulation of Innate Immunity

That activity of innate immune cells is regulated by activation of ARs has been clearly demonstrated. Adrenergic signaling affects macrophage polarization and cytokine production. β-AR signaling promotes macrophage differentiations toward an M2 phenotype which produces anti-inflammatory cytokines (42–45). In one study, gut macrophages in the muscularis mucosa, which are in close proximity to the autonomic myenteric plexus, differentially expressed the β2-AR, which mediated alternative activation of these macrophages, resulting in an M2 anti-inflammatory and tissue-protective profile (46). Both *in vitro* and *in vivo* studies support the conclusion that at least one way in which β-adrenergic signaling can promote breast cancer progression is by polarizing macrophages toward an M2 phenotype (42, 43). Moreover, in response to LPS stimulation, human monocyte-derived macrophages produce reduced amounts of the inflammatory cytokines TNF-α, IL-1β, CCL2, CCL3, and CCL4 (47–49) and decrease IL-27 secretion in response to acute inflammation (50) while, at the same time, increasing production of the anti-inflammatory cytokines IL-4, IL-10, and IL-13 production (44, 50). In contrast to the effects of β-AR signaling in macrophage, α-AR signaling promotes secretion of pro-inflammatory cytokines (24, 51).

Adrenergic signaling has been shown to impact DCs by impairing their maturation, cytokine production, and antigen presentation. Studies have shown that β2-AR activation prevents differentiation of monocytes into DCs (52) and enhances production of the anti-inflammatory cytokines IL-6, IL-10, and IL-33 while decreasing IL-12 and TNF-α production (53–58) by inhibition of NF-κB and AP-1 (54). In addition to altering cytokine production, Epi has also been shown to activate β1-AR/arrestin2–PI3K–MMP9/CCR7 signaling to inhibit migration of human DCs (59) which could impair migration to lymph nodes. Pretreating bone marrow derived DCs (BMDC) with β2-AR agonists before adoptive transfer reduces migration and responses to chemokines. Both *in vitro* and *in vivo* studies show that α2-AR suppresses DC migration by inhibiting type IV collagenase/gelatinase activity (60). The primary function of DCs, antigen presentation, is impaired by treating epidermal Langerhans cells with catecholamines, and this effect is blocked by using a β2-AR antagonist (61). In addition, cross-presentation of proteins by DCs is impaired by activation of β2-AR signaling, which in turn decreases CD8^+^ T cell proliferation and IL-2 production (62). Our lab has also found that adrenergic stress impacts the phenotype and function of DCs (63) in ways that could suppress their function.

Several studies have demonstrated that chronic adrenergic signaling can suppress NK cell activity. In a restraint stress mouse model, the number of NK cells is reduced in the intraparenchymal region of the lung and circulation (64). Another study showed that activation of β-ARs reduces the activity (65) of murine NK cells and leads to an increase in tumor metastasis. Interestingly, the two arms of the stress response can cooperate to regulate immune cells. De Lorenzo et al. found that during sleep deprivation, glucocorticoids increase expression of β2-AR in NK cells resulting in reduction of NK cell numbers and cytotoxicity (66). In contrast to the effects observed in these models of chronic stress, some studies have concluded that stress can *increase* NK cell activation and function. In one such study, six episodes of social disruption increased NK cell activity (67). In addition, voluntary exercise reduces the incidence and growth of breast cancer and this effect is mediated by catecholamine induced plasma IL-6, which in this case mobilizes NK cells (17). Enriching the housing environment for mice also increases NK antitumor function (68). Clearly, the effects of stress differ depending on the type and duration.

In addition, there are many reports investigating the regulation of other innate immune cells by adrenergic signaling. For example, a β2-AR agonist was able to prevent eosinophil functions which are induced by exposure to IL-5, LTD4, or IP-10 and which worsen the severity of asthma (69). In another example, the phagocytic efficiency of wound neutrophils was found to be impaired by a pharmacologic dose of NE, in a standard subcutaneous sponge wound model, and this change was found to be mediated by α- and β-ARs and downstream protein kinase A (70).

### Adrenergic Regulation of Adaptive Immunity

Norepinephrine also activates ARs on lymphocytes to regulate the differentiation, trafficking/migration, and effector functions of all lymphocyte subpopulations (11). Adrenergic signaling impacts T-cells by directly regulating thymocytes and inhibiting activation, differentiation, and effector function of T-cells directly or indirectly by inhibiting T-cell activating cytokine production by DCs. A study in stressed mice showed that β-AR signaling promotes negative selection in the thymus through the p38 mitogen-activated protein kinase pathway, resulting in decreased numbers of thymocytes (71). In addition, exposure to chronic unpredictable stress reduces the number of double negative thymocytes (72). Adrenergic signaling decreases production of IL-2, IFN-γ, and proliferation of CD4^+^ T-cell through many mechanisms. β2-AR signaling inhibits cytokines by inhibiting calcineurin in a PKA dependent manner (73). IFN-γ production by CD4^+^ T-cells can also be affected indirectly by inhibition of cytokine production by DCs. Adrenergic signaling also regulates Th1 and Th2 differentiation (34). Naïve CD4^+^ T-cells or activated Th1 cells express a detectable level of the β2-AR, while Th2 cells do not, due to differences in histone and DNA modifications within the β2-AR proximal promoter (22, 74). Therefore, Th1 cells can be suppressed by adrenergic signals and β2-AR activation has been found to be involved in directing CD4^+^ T-cell polarization toward a Th2 phenotype (75). The impact of β2-AR signaling on CD8^+^ T-cells depends on the stage of differentiation. Memory and effector CD8^+^ T-cells have significantly higher expression of β2-ARs than naïve T-cells and β2-AR signaling reduces IL-2 and IFN-γ production by memory CD8^+^ T-cells upon restimulation (76). IL-2 increases β2-AR expression on effector CD8^+^ T-cells; however, β2-AR signaling suppresses IL-2 production by CD8^+^ T-cells, which forms a negative-feedback loop in regulating CD8^+^ T-cell activation and function (77). By contrast, while β2-AR signaling suppresses CD8^+^ effector T-cell function, β2-AR signaling in regulatory T-cells increases their suppressor activity by increasing expression of the checkpoint molecule CTLA-4 and promotes inducible Tregs by inducing Foxp3 expression in T-cells (55).

Suppression of immune cells by chronic adrenergic stress has long-term negative implications for disease progression and has been well studied in the context of both infectious diseases and autoimmunity. β2-AR signaling impairs CD8^+^ T-cell-mediated antiviral responses to influenza *in vivo* and blocking the β2-AR with Nadolol enabled development of a robust antiviral response (78). Similarly, β2-AR signaling reduced the CD8^+^ T-cell response to the vesicular stomatitis virus (79). Dysregulation of the immune response also underlies many autoimmune diseases. Because autoimmune diseases involve overactive immune responses, adrenergic signaling actually helps to suppress autoimmunity, and ablation of sympathetic nerves worsened disease severity in a mouse model of multiple sclerosis (80). An in depth discussion of these studies is beyond the scope of this review but is the subject of a recent review by our group (81).

## Neural Regulation of Tumor Growth

The early observations of Levi Montalcini and collaborators first clearly demonstrated that tumor cells secrete nerve growth factors and can induce neural outgrowth from both sensory and sympathetic ganglia (82). As evidence of the tumor promoting effects of adrenergic signaling accumulated, Entschladen and colleagues hypothesized that in a process analogous to angiogenesis and lymphangiogenesis, tumors might secrete neurotrophic factors to attract their own innervation (83). But it was the elegant study of Magnon et al. (84) that clearly demonstrated both the ability of a developing tumor to attract autonomic innervation and the critical role such innervation plays in development and progression of a tumor. Using an orthotopic xenograft model (PC-3 prostate cells), they showed that the developing tumor attracted new autonomic innervation and that if they severed the hypogastric nerves or chemically sympathectomized the mice, tumor growth was significantly inhibited. Furthermore, they showed that parasympathetic nerves promoted the later stages of invasion and metastasis. Others have shown that NE levels in tumors are elevated by stress that activates the SNS and that NE is produced locally within tumors (as evidenced by tyrosine hydroxylase positive cells) (81, 85–88).

**Figure 1:**
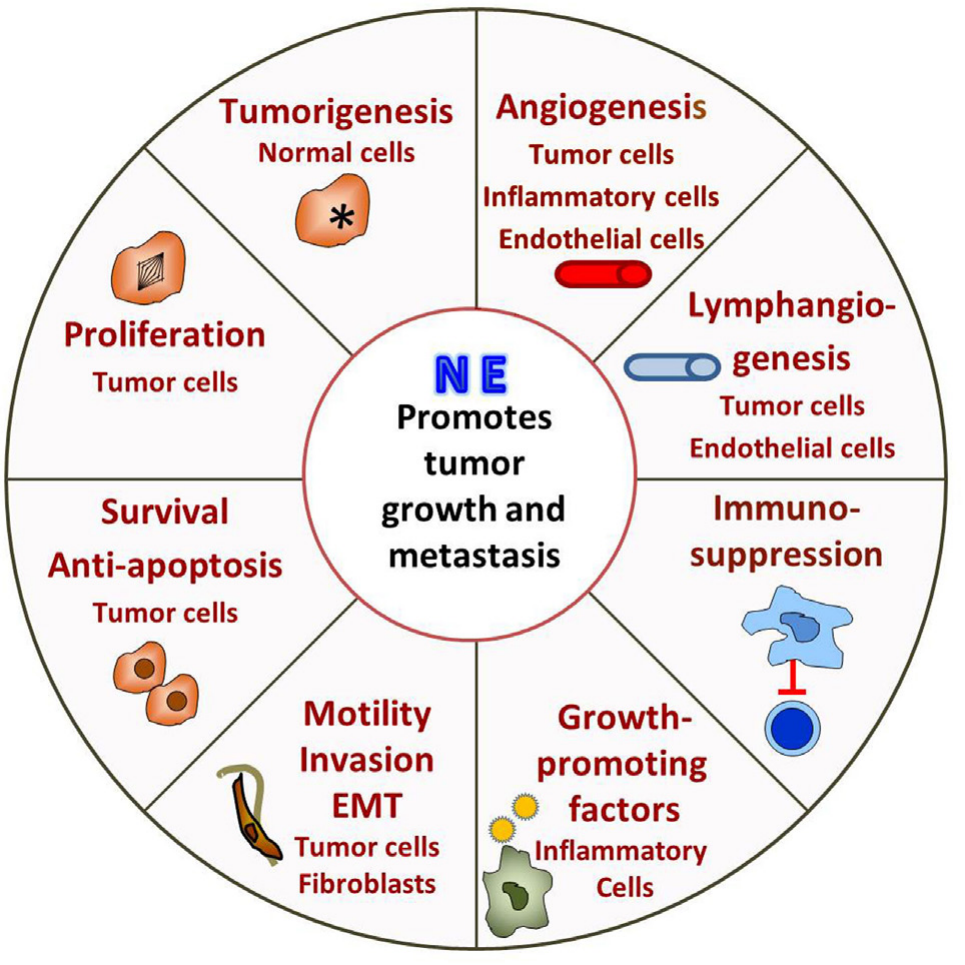
Adrenergic signaling promotes tumor survival, growth, and metastasis. The tumor is innervated by postganglionic nerves of the sympathetic nervous system and, in response to stress, these nerves secrete norepinephrine (NE). Many cells in the tumor microenvironment express adrenergic receptors, and their responses support tumor growth. See text for discussion.

In the last decade or so there has been a growing effort to understand how adrenergic signaling promotes tumor growth and we now know that β-AR signaling acts through multiple mechanisms (Figure 1). ARs are expressed by multiple cell types found in the tumor microenvironment and, therefore, the effects of adrenergic signaling that support tumor survival, growth, and metastasis are complex.

Direct effects include promoting tumorigenesis, tumor cell proliferation, antiapoptotic mechanisms, and promoting metastasis by inducing epithelial to mesenchymal transformation (EMT), motility and invasion. β-Adrenergic signaling activates p53 degradation and DNA damage through the β-AR/ARRB1/PKA pathway (89–91) and activation of oncogenes, such as Src and Her2 (92) and may promote tumorigenesis. In addition, increased psychological stress is associated with decreased telomere length (93), which is associated with increased cancer risk (94). Adrenergic signaling promotes tumor cell proliferation both *in vitro* and *in vivo* (95–101), while blocking adrenergic signaling leads to G1/S phase cell cycle arrest and apoptosis (102, 103). Antiapoptotic mechanisms are upregulated in tumor cells both *in vitro* and in mouse xenografts (84, 85, 104–106). Our lab found that adrenergic signaling increases expression of the antiapoptotic proteins BAD, BCL-2, and MCP-1 in tumors and blocking adrenergic signaling significantly reduces antiapoptotic protein expression and tumor growth (85). In addition, *in vitro*, NE activates the TGF-β pathway in treated cancer cells enhancing migration and invasion (107, 108). Mesenchymal markers such as α-SMA, vimentin, and snail are also increased by β-adrenergic signaling (109, 110). More recently, analysis of exosomes isolated from ovarian patients with high levels of adrenergic pathway activation (associated with low social support) shows upregulation of mesenchymal-characteristic gene transcripts and downregulation of epithelial-characteristic gene transcripts, and this is mediated by the ADRB/cAMP/PKA pathway (111). Adrenergic signaling plays a vital role in promoting of metastasis (112–122) and could directly induce invasive genes such as MMPs by tumor cells (123) and indirectly by causing cancer-associated fibroblasts to produce high levels of collagen and extracellular matrix components, which facilitate tumor invasion and dissemination (124).

Adrenergic signaling also affects other cells in the tumor microenvironment, which in turn support tumor growth, survival and metastasis. Studies show that adrenergic signaling promotes angiogenesis through increasing VEGF, IL-8, IL-6, PEG2 expression in tumor cells (87, 119, 125–130), and decreasing expression of TSP1, a potent angiogenesis inhibitor (129). A recent study from Zahalka et al. demonstrates that adrenergic signaling also directly alters the metabolism of endothelial cells in the TME, leading to increased angiogenesis (131). In addition, studies demonstrate that adrenergic signaling promotes lymphangiogenesis by inducing VEGFC production by tumor cells and/or macrophages (112, 132). Formation of a pre-metastatic niche in the lung is induced by adrenergic signaling in monocyte/macrophage cells in a breast cancer model (133). In another study, stimulation of bone marrow stromal cells by adrenergic signaling increases breast cancer bone colonization (115). Finally, it is becoming clear that the immunosuppressive effects of adrenergic signaling that have been seen in infection models (78, 79) are also playing a major role in suppressing the antitumor immune response. This inhibits tumor infiltration and function of cytotoxic T-cells (134–136) and promotes infiltration of immunosuppressive cells such as MDSC and Tregs (134, 136–139).

## Modeling the Tumor-Promoting Effects of Adrenergic Stress/Signaling in Mice

Although the concept of stress is generally understood, it is challenging to define. The original concept of stress, as proposed by Selye, was the response of an organism to a stimulus that challenged its well-being and he called this the General Adaptation Syndrome (140). Briefly, he proposed that an organism had the same response to many different noxious stimuli (swelling of the adrenal cortex, atrophy of the thymus, gastric ulcers) and that people suffering from various diseases had the same symptoms or syndrome. Eventually, he coined the term “stressor” to refer to the stimulus and the “stress response” to refer to the reaction. Stressors can be psychological (such as anxiety, fear, depression), physical (such as injury, surgery), or physiological (environmental factors including temperature) and exposure to stressors can be either acute (a single short-term exposure—minutes to hours) or chronic (long term, ongoing, or repeated). In general, an acute stress such as an injury leads to a beneficial response including activation of immune cells, whereas chronic exposure is most often deleterious and immunosuppressive (141). Dhabhar (18) has discussed the idea of categorizing stress as “protective, pathological, or regulatory” depending on their effects on overall health.

There is a general view that stress is detrimental to health and in ovarian carcinoma patients with little social support, it has been shown that the stress of social isolation elevates tumor and ascites NE while Epi was undetectable; this elevated NE correlates with more severe disease stages (86). To study how stress impacts tumor growth in preclinical models, protocols for inducing stress in mice have been developed (Table 1). It is important to point out that the same method can be used to induce both acute and chronic stress and these are distinguished by the duration of exposure. A good example of this is a study that used restraint stress to compare changes in the hippocampal transcriptome following acute vs. chronic stress by looking at samples taken after 1, 8, or 13 days of stress exposure (142).

**Table 1:** Effects of different models of stress on tumor growth in mice.

Reference	Tumor model	Effects on tumor growth	Mechanisms
**Psychological stress—restraint: confinement, unable to move freely**			
Kim-Fuchs et al. (123)	Human pancreatic cancer/nude mice	Increases tumor growth and dissemination of tumor cells to adjacent pancreas and liver	β-Adrenergic receptor (AR) signaling induces expression of tumor invasion genes and matrix metalloproteases, MMP2 and MMP9
Le et al. (132)	Human breast cancer/nude mice Murine breast cancer/BALB/c MMTV-PyMT-C57BL/6	Promotes lymphangiogenesis and tumor cell dissemination and metastasis	β-AR induces tumor-associated macrophages producing inflammatory molecules such as PGE2, which in turn induce tumor cells to produce VEGFC promoting lymphatic remodeling
Hulsurkar et al. (129)	Human prostate cancer/NOD/SCID	Promotes tumor growth	β-AR signaling activates CREB and induces HDAC2 expression by binding to its promoter. HDAC2 repression of TSP1 expression, promotes angiogenesis and prostate cancer progression
Hassan et al. (104)	Prostate cancer/nude mice Hi-Myc mice (and fear)	Antiapoptotic effect on tumor	Increased tumor catecholamine levels, which activates the epinephrine/ADRB2/PKA/BAD antiapoptotic signaling pathway
Nagaraja et al. (119)	Human ovarian cancer injected intraperitoneally or into ovaries (metastasis model)/nude mice	Increases tumor production of inflammatory prostaglandins and tumor metastasis	Increases prostaglandin E2(PGE2) synthesis *via* ADRB2–NF-κB–PTGS2 axis
Nagaraja et al. (124)	Human ovarian cancer/nude mice Murine ovarian cancer/C57BL/6	Increases tumor growth	NE drives cancer-associated fibroblast (CAF) phenotype *via* ADRB2/CREB/INHBA axis. CAFs produce high levels of collagen and extracellular matrix components
Lin et al. (143)	Human colon cancer/nude mice	Increases tumor weight	Increases plasma catecholamine; induces hyper-phosphorylation of ERK1/2, which drives cell proliferation
**Psychological stress—social isolation: individual housing**			
Thaker et al. (88)	Human ovarian cancer/nude mice (and restraint stress)	Increases tumor burden; more invasive growth of tumor	Increases size of adrenal glands; higher levels of tissue catecholamine; enhances tumor angiogenesis and enhances tumor expression of VEGF, MMP2, and MMP9 by activation of ADRB2/cAMP/PKA pathway
Madden et al. (144)	Human breast cancer/SCID	Increases tumor growth	Increases tumor F4/80^+^ and CD11b^+^Gr-1^+^ macrophage populations
Chen et al. (133)	Murine breast cancer i.v./BALB/c; MMTV-PyMT-C57Bl/6 (and chronic unpredictable stressors^a^)	Promotes breast cancer metastasis to lung	β-AR signaling induces expression of CCL2 in pulmonary stromal cells and CCR2 in monocytes/macrophages; increases recruitment and infiltration of macrophages into the pre-metastatic lung
Qin et al. (42)	Murine mammary cancer/BALB/c	Increases tumor growth	Increases serum catecholamine levels; increases migration of 4T1 cells *in vitro*; polarizes macrophage to M2 phenotype
**Psychological stress: acoustic**			
Hou et al. (145)	Murine colon cancer/BALB/c	Promotes tumor progression	Increases serum catecholamine and corticosterone; change Th1 and Th2 cytokines, and shift from Th1 to Th2 response in both circulation and tumor
Partecke et al. (146)	Murine pancreatic cancer/C57BL/6	Increases tumor growth and reduces survival	Increases behave stress; increases serum corticosterone and adrenal tyrosine hydroxylase; reduces Th1 cytokines; increases infiltration of Treg cells in tumor; increases VEGF and TGF-β with greater microvessel densities; increases MMP9 expression
**Physical/psychological stress: surgery**			
Lee et al. (147)	Human ovarian cancer/nude mice	Increases tumor growth	Increases angiogenesis; increases serum G-CSF, IL-1a, IL-6, and IL-15 concentrations
**Physical/physiological stress: housing temperature-induced stress: standard housing at 22°C vs. housing at thermoneutrality 30°C**			
Eng et al. (85)	Human pancreatic cancer/SCID Murine pancreatic cancer/C57BL/6	Increases tumor antiapoptosis, resistance to chemotherapy, tumor growth	Increases tumor catecholamines; increases antiapoptotic proteins expression
Bucsek et al. (134)	Murine mammary cancer/BALB/c Murine melanoma/C57BL/6	Increases tumor growth	Increases serum catecholamine; decreases tumor infiltrating CD8^+^ T cells and CD4^+^ T cells
Kokolus et al. (136)	Murine mammary cancer, colon cancer/BALB/c Murine melanoma/C57BL/6	Increases tumor growth	Decreases tumor infiltrating CD8^+^ T cells, CD4^+^ T cells; increases immunosuppressive cells
**Acute stress: restraint**			
Dhabhar (18)	Ultraviolet-B (UV) induced squamous cell carcinoma	Decreases tumor incidence and fewer tumors	Increases cutaneous-T-cell-attracting-chemokine (CTACK)/CCL27, RANTES, IL-12, and IFN-γ gene expression; increases skin infiltrating T cell numbers
**Exercise stress: voluntary running**			
Pedersen et al. (17)	Melanoma or lung cancer/C57BL/6 DEN-induced liver tumors/NMRI male mice	Reduces tumor incidence and growth	Increases serum catecholamine; increases plasma IL-6: mobilizes NK cells
Dethlefsen et al. (148)	Stage I/II breast cancer patient; human breast cancer/female NMRI-Foxn1nu mice	Reduces tumor growth	Exercise-conditioned human serum decreases breast cancer cell viability and tumorigenic potential; catecholamine induces Hippo tumor suppressor signaling pathway, which inhibits tumor growth
**Environmental enrichment^b^**			
Song et al. (68)	Murine pancreatic cancer and lung cancer/C57BL/6/Beige mice/Rag1^−/−^ mice	Decreases tumor growth and benefit is lost if mice receive a β-blocker or chemical sympathectomy	β-Adrenergic signaling enhances NK cell-mediated antitumor immune responses; increases expression of CCR5 and NKG2D in NK cells; and increases tumor infiltration of NK cells

*^a^Chronic unpredictable stressors include cage tilt, isolation, crowding, rapid light–dark changes, damp bedding, and overnight illumination*.

*^b^Environment enrichment: mice housed 12/cage in large cages with running wheels, tunnels, wooden toys, small huts, and nesting materials, which were moved 2×/week and changed 1×/week*.

To model psychological stress, mice have most often been subjected to social isolation or restraint stress. Over 10 years ago, using a mouse xenograft model of ovarian cancer, Thaker’s group showed that restraint stress resulted in significant elevation of NE (255–358%) and corticosterone (488–789%) in organs adjacent to the peritoneal cavity in tumor-bearing mice. Tumor growth and metastasis were also significantly promoted in these mice. Mice are social animals and are normally housed at four to five per cage; in fact, special permission is needed if mice are going to be housed singly and in that case, enrichment materials are required. In addition to restraint stress, Thaker’s group also subjected mice to social isolation and found enlargement of the adrenal glands as well as confirming the tumor promoting effects of stress. In addition, they showed that these effects could be duplicated by treating mice with the β2-AR *agonist* terbutaline and that the tumor promoting effects of both restraint and social isolation stress could be blocked by the pan β-AR *antagonist* propranolol. Altogether, these results clearly defined a role for adrenergic signaling in tumor growth and this was shown to be mediated by tumor VEGF production and increased angiogenesis (88). Since these seminal studies, the role of adrenergic signaling in tumor progression has been characterized in other models. Other groups have noted increased levels of catecholamines in response to psychosocial stressors. Qin et al. found that Epi was elevated and breast cancer progression was promoted in association with an M2 skewed macrophage population in a social isolation model; however, NE levels were not reported (42). Partecke et al. found that in response to a combination of acoustic and chronic stress, animals’ measures of behavioral stress as well as levels of stress hormones (steroids and adrenal tyrosine hydroxylase, the rate limiting enzyme in NE synthesis) were significantly elevated and furthermore, that orthotopic, syngeneic pancreatic tumors grew faster in these mice and there were indications that their immune response was suppressed, with a trend toward fewer CD4 and increased intratumoral Tregs present (146). Treatment of these mice with propranolol reduced tumor growth and improved overall survival (OS). These studies and several others (see Table 1) demonstrate that psychosocial stress promotes tumor growth through various mechanisms and that adrenergic signaling can be blocked by administration of β-AR or specific β2-AR antagonists.

In laboratory studies, the effects of *physical/physiological* environmental stressors on catecholamine levels and tumor growth have not been as well studied as the effects of psychological stresses, but environmental factors have great capacity to induce a stress response and alter internal metabolism and physiology (149). Our lab has recently defined a role for chronic mild cold stress in promoting tumor growth. This environmental stressor is universally imposed on mice as a result of mandated, cool ambient housing temperatures (150), and the impact of this cold stress on the modeling of several non-tumor mouse models of disease has been recently reviewed (151–153). The increasing awareness of this problem is reflected in several additional publications over the last 2–3 years reporting on how ambient housing temperatures affect disease outcomes in atherosclerosis (154–157), Alzheimer’s (158), monocyte mobilization into the blood (159), and obesity (160–162). Newer reviews have also drawn attention to this situation (163, 164). We first reported the unexpected discovery that when mice are housed at thermoneutrality (30°C), tumor growth is significantly inhibited in comparison to mice housed at standard temperatures (22°C) even though mice under both conditions maintain a normal core body temperature of 37°C (136). We also demonstrated that this difference in tumor growth was dependent on the adaptive immune response which was significantly suppressed when mice were housed at 22°C. Furthermore, we found that although the effects of cold stress are commonly studied by exposing mice to 4°C, just housing mice at the sub-thermoneutral 22°C was sufficient to cause chronic cold stress and significant elevation of both plasma and tumor NE levels (85, 134). Thus we found that even what was considered mild cold stress at 22°C, is biologically sufficient to induce SNS activation to produce NE and drive non-shivering thermogenesis to maintain a normal body temperature. Furthermore, we have shown that chronic adrenergic signaling in mice housed at 22°C promotes tumor growth in two different ways, both by induction of antiapoptotic signaling molecules and resistance to cytotoxic therapies in tumor cells (85) and by profound immunosuppression of the antitumor immune response which is associated with increases in immunosuppressive cell populations and inhibition of CD8^+^ T-cell effector phenotypes (134). Therefore, not only are the tumor cells more resistant to being killed, but the ability of the immune cells to kill tumor cells is much less robust when NE levels are increased by baseline housing stress; both of these situations are reversed by administration of the β-blocker propranolol. Interestingly, the recent studies of Wrobel et al. (135) agree with our findings; this group administered propranolol to MT/Ret mice and found inhibition of tumor development, increased CD8^+^ T-cells in the tumors, and decreased MDSC. However, these authors did not associate this baseline stress with housing temperature and the temperature at which their mice were housed was not reported.

We are particularly concerned about this immunosuppression by baseline adrenergic stress in experimental mice because both the long-term outcome of traditional therapies (165–170) and immunotherapies (171) depend on the development of a robust antitumor immune response. We tested whether the efficacy of immunotherapy, specifically checkpoint inhibitor therapies, was limited by adrenergic stress. In mouse models of melanoma and breast cancer, we used three separate approaches: housing mice at 30°C, treating mice housed at 22°C with β-blockers, or testing anti-PD-1 in ADRB2^−/−^ mice at 22°C and asked how this impacted in efficacy of anti-PD-1. In each case, it is clear that adrenergic signaling reduces responses to checkpoint inhibitor therapy and the efficacy is significantly improved by reducing this stress.

**Figure 2:**
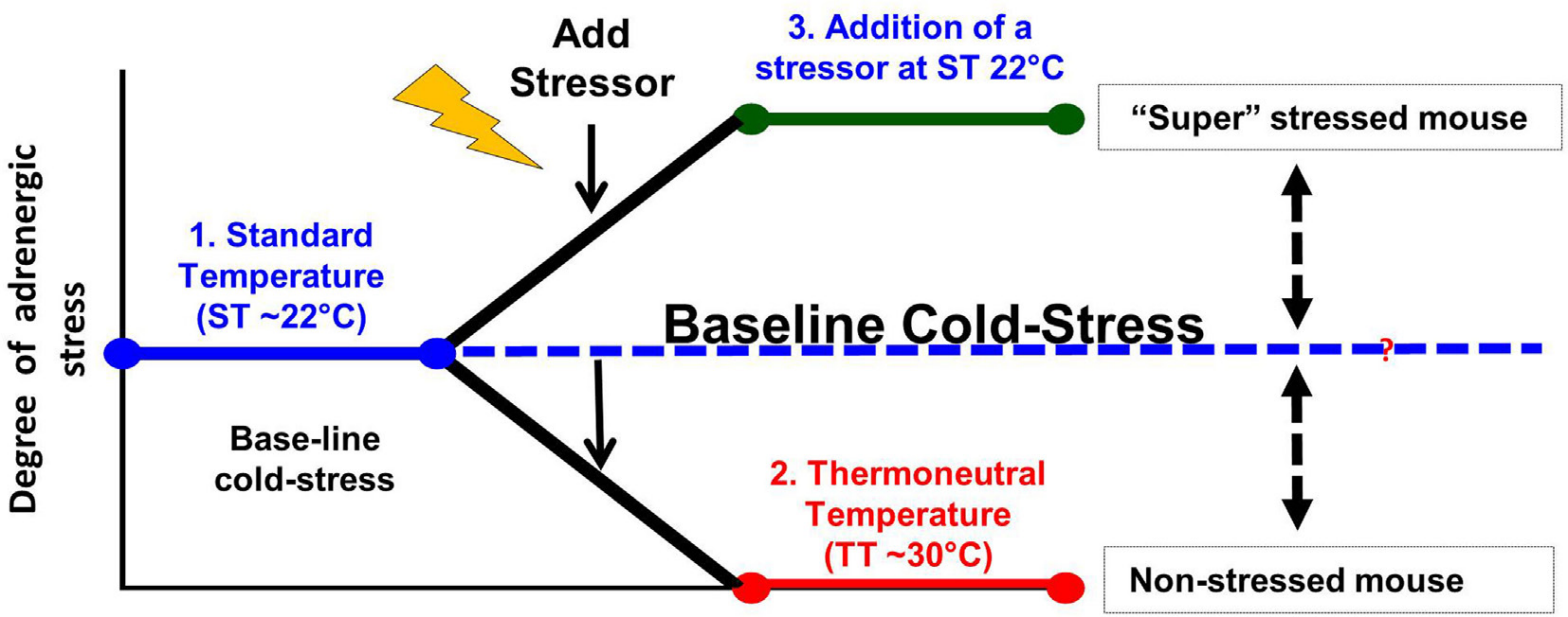
Modeling adrenergic stress in mouse models. 1. Mandated housing of mice at ~22°C imposes chronic cold stress and results in elevated norepinephrine (NE) levels which drive thermogenesis to maintain a normal body temperature of 37°. This “baseline cold stress” is sufficient to inhibit the development of an immune response in several disease models including cancer. 2. Reduction of NE levels and reversal of immunosuppression can be achieved by housing mice at thermoneutrality (~30°C) or administration of β-blockers. Reduction of baseline adrenergic stress significantly improves the antitumor immune response in preclinical models compared with the response in mice housed at 22°C. The improved immune response correlates with a significant improvement in the efficacy of immunotherapy. 3. The majority of studies compare the impaired immune response seen at 22°C with that observed after an additional source of stress is imposed on animals at 22°C. This approach may compromise a full understanding of the capabilities of the endogenous immune response and could also lead to a misunderstanding of the efficacy of therapies depending on an immune response in these models.

Taken together, it is clear that the majority of studies on the effects of stress are performed at 22°C when the “control” mice are already “stressed” in comparison to the relatively reduced levels they would be experiencing at 30°C (Figure 2). Therefore, restraint stress or other forms of psychosocial stress are imposed on already stressed mice, so that measures of tumor growth, response to therapy or the capabilities of the antitumor immune response in the controls are somewhat compromised. In the future, it will be important to determine if, and/or how, baseline cold stress may be affecting the results of these stress experiments. Although due to the thermal comfort requirements of personnel, it seems unlikely that the ambient temperatures in animal facilities will be changed to 30°C, it may be possible to reduce cold stress in experimental animals by providing nesting materials or constructing special cages in which mice can behaviorally regulate their thermal comfort (172–174).

## Clinical Relevance of Preclinical Findings

How do these findings relate to patients? Studies have demonstrated that several types of patient tumors express ARs including pediatric (175, 176), pancreatic (103), lung (177), melanoma (178), and prostate (179) cancers. More provocatively, as it became apparent that blockade of adrenergic signaling could inhibit tumor growth in preclinical models, researchers looked for evidence of benefit in patients. Several retrospective epidemiological studies have supported the conclusion that patients who are taking β-AR antagonists (“β-blockers”) and receiving conventional therapies have better outcomes in terms of both progression-free survival and OS than those who are not (Table 2). It should be noted, however, that there are other analyses that failed to identify any benefit of β-blockers (180–186). There are several potential reasons for why some studies show that cancer patients benefit from taking β-blockers while other studies conclude there is no benefit. One important reason is that the type of β-blocker being taken by the patient likely influences the patient’s response as well as the overall outcome of the study. There are studies showing that patients taking non-selective β-blockers (blockade of β1 and β2) had improved OS compared with patients taking β1-specific blockers which are the most commonly prescribed β-blockers (180, 183, 184, 187). The heterogeneity of ARs expressed by the tumors themselves may also account for the different responses in cancer patients (180, 184). Besides the type of β-blocker, it is possible that the dosage needed to elicit a favorable antitumor effect is higher than the normal prescription dose for treating cardiovascular diseases (182, 185). In addition, the patient parameters are variable among the studies. Although the most important potential confounders such as sex, stage, treatment were usually adjusted for, there are others, such as socioeconomic status which is proven to be associated with cancer progression, which were not taken into consideration (182, 185). The timing of β-blocker exposure is different between studies. Some studies recruited patients already taking β-blockers before cancer diagnosis, while some studies investigated patients who began taking β-blockers after cancer diagnosis (180, 181, 183, 184). In addition, there are limited records showing whether patients complied with the prescriptions (182, 185). The study design may also influence the result. Some studies used all-cause of mortality as the endpoint, which makes it hard to study the relationship of β-blocker usage and cancer-specific mortality (183). In other studies, patients taking β-blockers who died within 3 months after cancer diagnosis were still included, which is thought to be too short an interval to assess benefit (181–184).

**Table 2:** A summary of retrospective studies assessing the beneficial effects of β-blockers, including Propranolol, in patients with different cancers who were also taking β-blockers.

Reference	Cancer type	Patients (numbers)	Survival	OR/HR	95% CI	*P*	Therapeutic effect
**β-Blockers (other than propranolol)^a^**							
Grytli et al. (188)	Prostate cancer	263		HR: 0.14	0.02–0.85	0.032	Reduced prostate cancer-specific mortality
Grytli et al. (189)	Prostate cancer	3,561		ASR: 0.79	0.68–0.91	0.001	Reduced prostate cancer-specific mortality
Kaapu et al. (190)	Prostate cancer	24,657		OR: 0.73	0.56–0.96	0.038	Decreased risk of advanced prostate cancer
De Giorgi et al. (191)	Thick melanoma	121			0.11–0.54	0.002	Reduce risk of progression of thick malignant melanoma
Lemeshow et al. (192)	Malignant melanoma	4,179		HR: 0.87	0.64–1.20		Increase survival time of patients with melanoma
Diaz et al. (193)	Epithelial ovarian cancer	248	PFS OS	HR: 0.56		0.05 0.02	Reduce chance of death
Wang et al. (194)	Non-small-cell lung cancer	722	DMFS DFS OS	HR: 0.67 HR: 0.74 HR: 0.78		0.01 0.02	Improved DMFS, DFS, and OS 0.02
Botteri et al. (195)	Triple-negative breast cancer	800		HR: 0.42	0.18–0.97		Significantly decreased risk of breast cancer-related recurrence, metastasis, and breast cancer death
Melhem-Bertrandt et al. (196)	Triple-negative breast cancer	1,413	RFS	HR: 0.30	0.1–0.87	0.027	Improve relapse-free survival in all patients with breast cancer and in patients with triple-negative breast cancer
Powe et al. (197)	Breast cancer	466		HR: 0.291	0.119–0.715	0.007	Significantly reduces distant metastases, cancer recurrence, and cancer-specific mortality in breast cancer patients
Jansen et al. (198)	Colorectal cancer	1,975		HR: 0.50	0.33–0.78		Association with longer survival
Monami et al. (199)	Cancer	1,340		HR: 0.33		0.019	Reduce cancer risk
Lin et al. (200)	Cancer	6,771		HR: 0.74	0.63–0.87	<0.001	Reduced upper gastrointestinal tract and lung cancer risk
**Propranolol**							
Choy et al. (201)	Triple-negative breast cancer	1,029		HR: 0.51	0.23–0.97	0.041	Decreased establishment of brain metastasis
Barron et al. (202)	Breast cancer	5,801		HR: 0.19	0.06–0.60		Reduce breast cancer progression and mortality
Nkontchou et al. (203)	Hepatocellular carcinoma	291		HR: 0.25	0.09–0.65	0.004	Decrease hepatocellular carcinoma occurrence
Chang et al. (204)	Head and neck Esophagus Stomach Colon Prostate	24,238		HR: 0.58 HR: 0.35 HR: 0.54 HR: 0.68 HR: 0.52	0.35–0.95 0.13–0.96 0.30–0.98 0.49–0.93 0.33–0.83		Reduce cancer risk

*ASR, adjusted hazard ratio; DFS, disease-free survival; DMFS, distant metastasis-free survival; HR, hazard ratio; OR, odds ratio; OS, overall survival; PFS, progression-free survival; RFS, recurrence-free survival; 95% CI, 95% confidence interval*.

*^a^β-Blockers other than propranolol include pindolol, propranolol, timolol, carvedilol, labetalol, nadolol, oxprenolol, alprenolol, sotalol, acebutolol, atenolol, betaxolol, bisoprolol, metoprolol, nebivolol, celiprolol, esmolol, carteolol, penbutolol, and celiprolol*.

To date, there has been a single report of a prospective study treating melanoma patients with propranolol. In this study, De Giorgi and colleagues offered patients the choice of “off-label” treatment with propranolol and found an 80% risk reduction for recurrence for the 19 of 53 patients who chose to receive it (205). Ultimately, the ability of β-blockers to support the development of an antitumor immune response and to improve the efficacy of traditional and immunotherapies will have be determined in blinded, prospective clinical trials, but the results of this study in melanoma lend support for the “repurposing” of β-blockers in oncology.

β-Blockers have been used extensively in the clinic to treat patients with hypertension, angina and anxiety. In general, β-blockers have a good toxicity profile and are well tolerated (206); common side effects include nausea, vomiting, diarrhea, insomnia, weakness and fatigue. However, the safety of using of β-blockers in cancer patients needs to be considered. Although most cancer patients do not have hypertension and/or angina, they do often have increased levels of adrenergic stress and so in that way may be considered to have abnormal adrenergic signaling. In several studies in which cancer patients were taking β-blockers as an off-label treatment, either no drug related adverse effects were recorded (205, 207) or small number of patients had minor side effects which did not require discontinuation (203, 208, 209). However, an understanding of the comparative safety of β-blockers in combination with other cancer therapies remains to be evaluated in the context of clinical trials.

## Conclusion

Recently developed immunotherapies are showing great promise in cancer treatment, yet many tumor types are not responding and even in sensitive tumors such as melanoma and lung cancer, the majority of patients are not benefiting. Therefore, developing new strategies for improving the therapeutic efficacy of cancer immunotherapies is critical. Immunotherapies and traditional therapies that have an immune-mediated component depend on the development of a robust antitumor immune response, but immunosuppressive factors often limit this. Therefore, a promising strategy to improve response to immunotherapy is to develop additional approaches to reverse immunosuppression that can be used in combination therapies.

A newly identified mechanism of immunosuppression is by stress-induced sympathetic adrenergic signaling, which suppresses many aspects of the immune system including development, differentiation, activation, and function of many types of immune cells. Adrenergic signaling has been shown to inhibit immune responses in both autoimmune diseases and infection models. Sympathetic neurons innervate both primary and secondary lymphoid organs supporting the idea that adrenergic signaling can impact immune cells locally. Recently, it has become clear that adrenergic signaling also affects tumor progression. By using mouse models of both psychosocial and physical stress stress-induced adrenergic signaling was found to be tumor-promoting in many types of cancers. The accumulated evidence demonstrates that adrenergic stress regulates tumor growth directly through multiple mechanisms, including development, proliferation, and protection of tumor cells from treatments. Although less well studied, recent findings show that adrenergic signaling also impacts tumor growth indirectly by regulating antitumor immunity. Our lab recently demonstrated that chronic mild cold stress induces SNS activation which promotes tumor growth; blocking this stress by either housing mice at thermoneutral temperature or adding β-blockers slows tumor growth. These effects were found to be meditated by CD8^+^ T-cells. In addition, combining a pan-β-blocker with an immune checkpoint inhibitor (anti-PD-1) significantly increased the therapeutic efficacy of anti-PD-1. Because cancer patients who were already taking β-blockers were found to have better outcomes in several retrospective studies, the preclinical studies provide a rationale for testing this combination in prospective clinical trials. Finally, accumulating evidence shows that external environmental stressors, i.e., housing temperature, creates a level of baseline adrenergic stress that affects experimental outcomes in mouse models. In the future, when designing and analyzing experiments, it will be important to factor in this potentially confounding experimental variable to better understand the biology underlying the results and to improve rigor and reproducibility.

## Author Contributions

GQ, MC, MB, ER, and BH developed the concepts for this review and were involved in identifying the references and writing and editing the manuscript.

## Conflict of Interest Statement

The authors declare that the research was conducted in the absence of any commercial or financial relationships that could be construed as a potential conflict of interest.
